# Bayesian mapping of pulmonary tuberculosis in Antananarivo, Madagascar

**DOI:** 10.1186/1471-2334-10-21

**Published:** 2010-02-05

**Authors:** Rindra V Randremanana, Vincent Richard, Fanjasoa Rakotomanana, Philippe Sabatier, Dominique J Bicout

**Affiliations:** 1 Unité Epidémiologie, Institut Pasteur de Madagascar, BP 1274, Antananarivo (101), Madagascar; 2 Unité Biomathématiques et Epidémiologie, Laboratoire EPSP- TIMC-IMAG, UMR 5525, Ecole Nationale et Vétérinaire de Lyon, 1 Avenue Bourgelat, 69280, Marcy l'Etoile, France

## Abstract

**Background:**

Tuberculosis (TB), an infectious disease caused by the *Mycobacterium tuberculosis *is endemic in Madagascar. The capital, Antananarivo is the most seriously affected area. TB had a non-random spatial distribution in this setting, with clustering in the poorer areas. The aim of this study was to explore this pattern further by a Bayesian approach, and to measure the associations between the spatial variation of TB risk and national control program indicators for all neighbourhoods.

**Methods:**

Combination of a Bayesian approach and a generalized linear mixed model (GLMM) was developed to produce smooth risk maps of TB and to model relationships between TB new cases and national TB control program indicators. The TB new cases were collected from records of the 16 Tuberculosis Diagnostic and Treatment Centres (DTC) of the city from 2004 to 2006. And five TB indicators were considered in the analysis: number of cases undergoing retreatment, number of patients with treatment failure and those suffering relapse after the completion of treatment, number of households with more than one case, number of patients lost to follow-up, and proximity to a DTC.

**Results:**

In Antananarivo, 43.23% of the neighbourhoods had a standardized incidence ratio (SIR) above 1, of which 19.28% with a TB risk significantly higher than the average. Identified high TB risk areas were clustered and the distribution of TB was found to be associated mainly with the number of patients lost to follow-up (SIR: 1.10, CI 95%: 1.02-1.19) and the number of households with more than one case (SIR: 1.13, CI 95%: 1.03-1.24).

**Conclusion:**

The spatial pattern of TB in Antananarivo and the contribution of national control program indicators to this pattern highlight the importance of the data recorded in the TB registry and the use of spatial approaches for assessing the epidemiological situation for TB. Including these variables into the model increases the reproducibility, as these data are already available for individual DTCs. These findings may also be useful for guiding decisions related to disease control strategies.

## Background

Tuberculosis (TB), the seventh most common cause disease in the world [1], is the leading cause of death from a curable infectious disease. In Madagascar, 18,000 to 20,000 new TB cases are detected every year [2]. The estimated incidence of pulmonary TB in the city of Antananarivo in 2004, 141 cases per 100,000 inhabitants, is the highest rate in the country [3].

The dynamics of infectious diseases depends on the spatial distribution of pathogens and hosts, and the probability of an encounter between them. The transmission of infectious pathogens from infected to susceptible hosts declines with increasing distance between individuals. TB, like many infectious diseases, is prone to spatial aggregation or clustering [4,5]. However in large cities, which tend to be overcrowded, with many highly mobile individuals, the spatial correlation generated by the transmission of infection may be disrupted, depending on the degree of mixing of the population [6]. A recent study based on a spatial scan statistic method showed that TB cases were highly concentrated in many neighbourhoods in Antananarivo [7]. Here, we re-examine this finding using a Bayesian approach, as previously described for analyses of the spatial distribution of prostate cancer incidence [8], lung cancer [9], malaria [10], giardiasis [11], equine infectious anaemia in horses [12]; modelling the effects of indicators of tuberculosis [13], schistosomiasis [14] and predicting the spatial distribution of schistosomiasis [15]. Previous disease mapping work was based on collating, mapping and analysing prevalence or incidence data with conventional statistical approaches, which are affected by random variation due to population variability and a loss of statistical power when cases are assigned to subgroups (e.g., several geographic subareas). Differences in geographic distribution due to chance may be incorrectly interpreted as true variation of epidemiological interest. The observed extreme values may not reflect the true spatial distribution of the disease, instead reflecting those of the population area. The Bayesian method can overcome these problems as it can model the random and true variation separately [16] and is an attractive alternative to the frequentist approach. Bayesian methods can provide some shrinkage and spatial smoothing of raw standardized incidence ratio estimates, which are strongly influenced by the size of the population at risk, resulting in a noisy and blurred picture of the true unobserved risks [17].

Tuberculosis is a disease with a social dimension, as it is known to be linked to socio-economic status. In developing countries, constraints on resources limit the collection of socio-economic data, which requires specific and costly investigation. In this study, we analysed the spatial distribution of pulmonary tuberculosis (TB) in Antananarivo and investigated the relationships between the indicators or outcomes and covariates included in the TB registry and TB incidence rates estimated from epidemiological data. We used indicator and covariate data from the national TB control program incorporated into a TB registry. These variables were related to both TB transmission and healthcare system quality. In this study, we investigated whether the spatial pattern of TB could be explained by considering indicators from the national control program without taking into account explicitly the socio-economic status of the concerned neighbourhood. To this end, we use a Bayesian approach to analyse data for new cases of TB reported during the period 2004-2006 in the city of Antananarivo, with the aim of increasing our knowledge of the true underlying geographic distribution of TB rates and improving prediction indicators.

## Methods

### Study area

This study was conducted in Antananarivo, the capital city of Madagascar. Antananarivo is the most densely populated city in Madagascar, with a population of 1,114,346 spread across 90 km^2^, giving a population density of almost 8687 inhabitants/km^2^. The city counts six administrative districts, comprising a total of 192 neighbourhoods, and is located at an altitude of 900 m to 1500 m and has a high-altitude tropical climate [18], with two seasons: a hot rainy season from April to October and a cold dry season from May to September. The average annual temperature is 18°C, with a maximum of 26°C (in November) and a minimum of 10°C (in July).

The population is served by three university teaching hospitals (*Centre Hospitalier Universitaire *(CHU)), 105 health centres and 16 Tuberculosis Diagnostic and Treatment Centres (DTC). The city has a good health service coverage but the neighbourhoods are considerably heterogeneous in terms of socio-economic conditions with some of them subjected to overcrowding, substandard housing conditions and unemployment.

### Epidemiological data sources

The 16 Tuberculosis DTC of the city provided the data for TB new cases. TB cases are registered in a routine information system as part of the official TB control program. The TB registry contains information on the patient's place of residence, treatment follow-up and status (new cases, retreatment, treatment failure, and relapse after the completion of treatment) and treatment outcome (recovery, completion of treatment etc). All cases were followed up during treatment, and new cases underwent bacteriological check-ups at 2, 5 and 7 months. Cases of pulmonary tuberculosis were defined as patients presenting a cough lasting for more than three weeks and confirmed by a positive sputum smear. All new cases recorded in DTC registries from 2004 to 2006 corresponding to patients resident in the city of Antananarivo were included in this study. Approval for this study was obtained from the National Ethics Committee of the Ministry of Health of Madagascar.

### Demographic and geographical data

Maps of the administrative districts and neighbourhood boundary lines were provided by the Development Office of Antananarivo (DOA). Population denominators for each neighbourhood were obtained from demographic census data for 2005.

### Bayesian approach

Combination of a Bayesian approach and a generalized linear mixed model (GLMM) was used to assess spatial heterogeneity in the TB standardized incidence ratio (SIR) and to investigate associations between the three- year average TB incidence rates and the following five variables: number of patients undergoing retreatment (*X*_*1i*_), number of patients with treatment failure and those suffering relapse after the completion of treatment (*X*_*2i*_), number of patients stopping treatment within two months (lost to follow-up) (*X*_*3i*_), number of households with more than one case (*X*_*4i*_), and distance from the patient's residence to the DTC (*X*_*5i*_). All these *X*_*ki *_(with *k = 1, 2, ..., 5*) , calculated for each neighbourhood "*i*" (*i *= 1, 2, ..., 192), were obtained from TB registries and incorporated into the national TB control program. These *X*_*ki *_were chosen as explicative variables because they are indicator of healthcare system and, to some extent, they carry more or less information on the socio-economic, hygienic status of the neighbourhood and therefore on the likelihood of TB transmission. The *X*_*5 *_is considered as a distal variable while the all others *X*'s as proximal variables for the TB transmission. For instance, as the TB is transmitted by close contacts between infectious peoples and susceptible ones, the number of households with more than one case can be informative of the population density, or one may wonder whether new cases are recruited from populations among which we find patients undergoing retreatment and/or with treatment failure. Likewise, the number of patients stopping their treatment may be indicative of their socio-economic conditions and/or education level, or living far away from the DTC could turn out to be penalizing for accessibility of health care facilities and thus discouraging patients to complete their treatment.

This study was conducted at the neighbourhood scale. For each neighbourhood "*i*" (*i *= 1, 2, ..., n), the expected number of new cases ε_i _was estimated as the mean new case rate over all districts multiplied by the population of the neighbourhood (i.e., ε_i _= mean new case rate × pop_i_), and the standardized incidence ratio (SIR) λ_i _of each neighbourhood "*i*" was calculated as the observed number of new cases divided by the number of expected cases. Within the Bayesian framework, the observed numbers of new cases y = (y_1_,..., y_n_) in the n neighbourhoods were treated as non-independent Poisson random variables with means μ = (μ_1_,..., μ_n_), where each μ_i _is given by μ_i _= ε_i _× λ_i, _or, in the logarithmic form, log(μ_i_) = log(ε_i_) + log(λ_i_). The SIR λ_i _is a function of the explicative variables *X*_*ki *_that account for differences and spatial heterogeneity in the disease rate: λ_i _= exp(β_0 _+ β_1_*x*_*1i*_*+ *β_2_*x*_*2i*_*+ *β_3_*x*_*3i *_*+ *β_4_*x*_*4i *_+ β_5_*x*_*5i+ *_*θ*_i _*+ ν*_i_) with *x*_*ki *_= *X*_*ki*_/SD_k_, where SD_k _is the standard deviation (over all neighbourhoods) of each variable.

For the β_k _we assumed non-informative Gaussian prior distributions with a mean of zero and a precision of 10^-5^, whereas β_0 _was assumed to have a flat distribution. In this context, *ν*_i _is a non-spatially structured random effect, assumed to have an independent Gaussian distribution of zero mean and variance σ^2^_ν _following an inverse Gamma distribution as 1/σ_v _~ dgamma(0.5, 5 × 10^-4^). This effect was generally included in the models to account for extra-Poisson variation due to important explicative variables that were not measured. The spatially structured random effects - θ = (θ_1_,..., θ_n_) - accounted for the spatial dependence, with the prior distribution taken as a conditional intrinsic Gaussian autoregressive model, in which the mean value for θ_i _is a weighted average of the neighbouring random effects and the variance, σ^2^_θ _following an inverse Gamma distribution of the form 1/σ_θ _~ dgamma(0.5, 5 × 10^-4^), controls the strength of this local spatial dependence, p(θ_i_/θ_j≠i_)~N(∑_*j≠i *_*w*_*ij *_θ_i_/∑_*j≠i *_*w*_*ij *_, σ^2^_θ_/∑_*j≠i *_*w*_*ij*_). As in most studies based on areas, we defined "neighbourhoods" as adjacent census tracts with simple binary adjacency weights, i.e. *w*_*ij *_= 1 if areas *i *and *j *share a common boundary and *w*_*ij *_= 0 otherwise.

These prior probability distributions and the likelihood of the data were updated and used in the Bayes' relation to obtain posterior distributions for the SIR [19]. The parameters were estimated by Markov chain Monte Carlo methods, using the public domain software package WinBUGS (Cambridge, UK) [20]. Two Markov chain Monte Carlo simulations were carried out in parallel, with different initial values, for parameter estimation. The time series plot for each parameter and Gelman-Rubin statistics showed that convergence occurred within 6,000 iterations. Thus, the inference of parameters was based on 20,000 iterations of both chains after the burn-in phase of 10,000 iterations. Each neighbourhood SIR was then input into a Geographic Information System for mapping. When investigating whether the posterior neighbourhood incidence rates were significantly higher or lower than the average rate, we have defined low risk (LR) and high risk (HR) neighbourhoods as follows. A HR neighbourhood was considered as having a rate significantly greater than the mean when the SIR was higher than 95% of iterations from the posterior distribution and SIR > 1. Likewise, a LR neighbourhood was considered as having a rate significantly lower than the mean when the SIR was higher than 95% of iterations from the posterior distribution and SIR < 1. In all other cases, the neighbourhood rate was considered to be not significantly different from the mean rate.

## Results

Antananarivo had 3075 notified new cases of TB during the study period, for which 2270 neighbourhoods of residence were identified. The poor quality of the patient address made the location assignment impossible for other patients.

Three separate models including/excluding the covariate/random effects were developed to investigate whether the covariates accounted for part or all of the spatial correlation in the TB SIR: a Bayesian model with spatial and non-spatial random effects only (model 1), a Bayesian model with covariates and non-spatial random effects (model 2), and a Bayesian model with both covariates and spatial and non-spatial random effects (model 3). The three models were quite similar on the basis of the deviance information criterion (DIC) only (see Table 1). However, model 2 had the highest variance of non-spatial random effects followed by model 1 and model 3 (see Table 1). The variance of the spatially structured effects, σ^2^_θ _was 0.0059 for model 1, which involved random effects but no explicative variables. The inclusion of explicative variables in model 3 decreased the variance of spatially structured effects to 4.6 × 10^-5^. Thus, model 3 was retained for the further analysis.

**Table 1:** Estimated Risk Factors Associated With TB Standardized Incidence Ratio by GLMM model, Antananarivo, Madagascar, 2004-2006

	Model 1: with spatial and non-spatial random effects only		Model 2: with covariates and non-spatial random effects		Model 3: with covariates, spatial and non-spatial random effects	
			
Covariates	β	SIR	β	SIR	β	SIR
Intercept	-0.055	-	-0.350	-	-0.289	-
Retreatment^a^	-	-	-0.032	0.968	-0.006	0.994
Relapse + treatment failure^b^	-	-	-0.012	0.988	-0.017	0.982
No households with case > 1^c^	-	-	0.134	1.143	0.127	1.135
Patients lost to follow-up^d^	-	-	0.101	1.106	0.099	1.104
Distance to DTC^e^	-	-	0.042	1.042	0.024	1.025
Non-spatial random effect variance	5.4 × 10^-4^		0.03		6.1 × 10^-5^	
Spatial random-effect variance	0.006		-		4.6 × 10^-5^	
Deviance Information Criterion(DIC)	960.7		958.5		961.4	

β: associated coefficient of the GLMM regression

SIR: standardized incidence ratio

^a^: number of patients undergoing retreatment/standard deviation of retreatment

^b^: number of patients with relapse + number of patients with treatment failure/standard deviation of relapse and treatment failure

^c ^:number of households with more than one case/standard deviation of households with more than one case

^d^: number of patients lost to follow-up/standard deviation of patients lost to follow-up

^e^: distance from patient's residence to DTC/standard deviation of patient's residence to DTC

**Figure 1:**
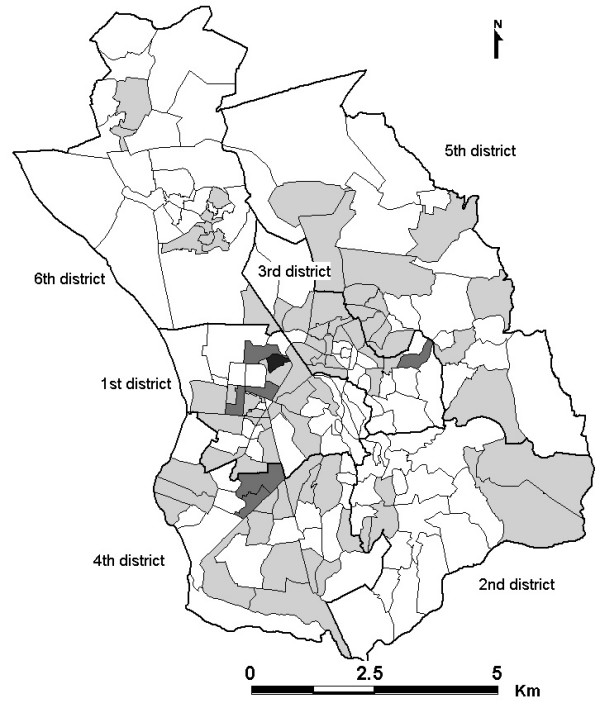
**Bayesian smoothed standardized incidence ratio for pulmonary TB diagnosed from 2004 to 2006 across Antananarivo neighbourhoods**. Mean standardized incidence ratio. "white square": <1. "pale grey square":1-2. "dark grey square":2-4. "black square": >4. "white rectangle with black bold outline": District boundaries. "white rectangle with black thin outline": Neighbourhood boundaries.

From model 3, the neighbourhood SIR ranged from 0.44 to 8 (maximal SIR = 18 × minimal SIR). The spatial distribution of the estimated SIR is displayed in Figure 1, where 83 out of 192 neighbourhoods (43.2%), scattered throughout the city, have SIR > 1. The variance of non-spatially structured effects which account for the intrinsic variability of SIR in each neighbourhood was 6.1 × 10^-5 ^(95% CI: 4.7 × 10^-7 ^- 0.05), and the variance of spatially structured effects was 4.6 × 10^-5 ^(95% CI: 2.7 × 10^-7 ^- 0.66).

**Figure 2:**
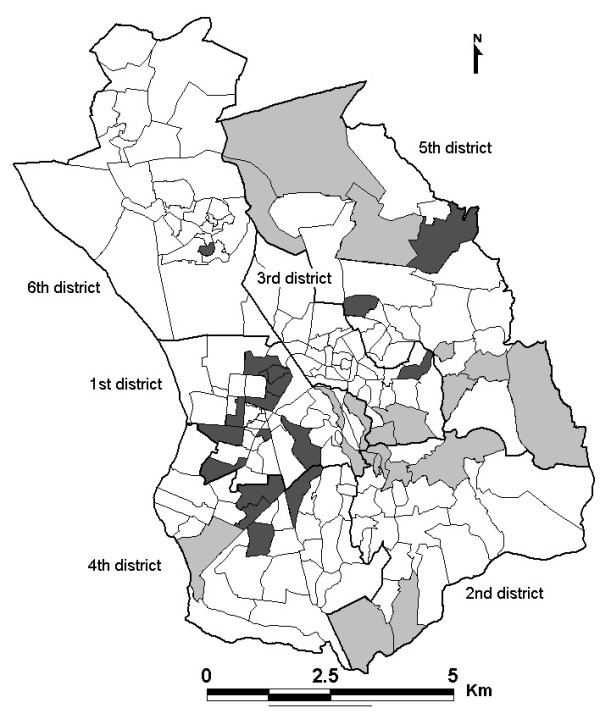
**Maps of significantly lower risk (LR) and higher risk (HR) neighbourhoods in Antananarivo, 2004-2006**. "white rectangle with black bold outline": District boundaries. "white rectangle with black thin outline": Neighbourhood boundaries. "pale grey square": Significantly lower risk neighbourhoods (SIR < 1 with high certainty). "dark grey square": Significantly higher risk neighbourhoods (SIR > 1 with high certainty).

As displayed in Figure 2, 21 LR (10.9%) and 16 HR (8.3%) neighbourhoods were identified. The 21 LR neighbourhoods had from 37% to 55% fewer cases than the average (case deficit), whereas the 16 HR neighbourhoods had from 55% to 713% more cases than the average (case excess).

**Figure 3:**
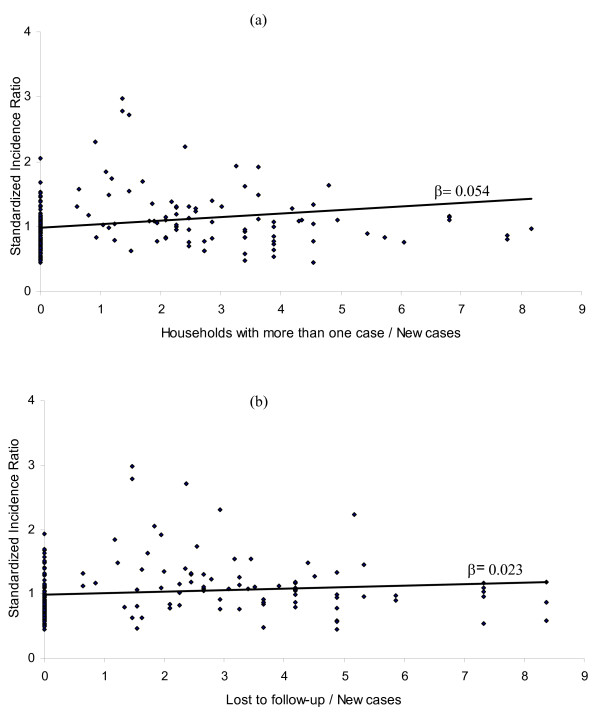
**Neighbourhood TB standardized incidence ratio in Antananarivo Madagascar, as a function of the 2 significant explicative variables, with linear regression model, 2004-2006**.

Parameter estimates for association between TB new cases and explicative indicators (proximal and distal variables for TB transmission) are reported in Table 1 (note that the β's are similar in both models 2 and 3). From model 3, patients living in households with more than one case were found to be at higher risk (SIR = 1.13, 95% CI: 1.03, 1.24) than those lost to follow-up (SIR = 1.10, 95% CI: 1.02, 1.19). As a check, these associations are plotted in Figure 3 (a, b) using a simple linear regression model.

## Discussion

We used a Bayesian approach to analyse the spatial distribution of pulmonary TB incidence in Antananarivo and to investigate association between TB rate and national TB control program indicators as explicative variables. Given that the DICs for the three models are not significantly different, we choose to retain the model 3 with the lowest variances of both random and spatially structured effects. As the variances of non-spatially structured and spatially structured effects both significantly decrease (by 89% and more than 100%, respectively) when incorporating explicative variables into model 1 to obtain model 3, this indicates that much of the dispersion and spatial pattern of the TB incidence are taken into account by explicative variables. This finding is also consistent with the fact that the spatial distribution of explicative variables was heterogeneous and clustered.

We have found at the neighbourhood scale that there is a strong geographic heterogeneity in pulmonary TB risk, with clusters of high risk areas, as previously reported [7], and two proximal variables the "number of household with more than one case" and "number of patients lost to follow-up" are the important explicative indicators of the TB distribution. Therefore, one may hypothesize that TB transmission in the context of Antananarivo would mainly originate from the clustered risk factors (like households with many cases and/or patients being lost to follow-up) rather than resulting from nearest neighbour diffusion. Confirmation of such a hypothesis will require genetic investigations (genetic investigation of *Mycobacterium tuberculosis *strains) and epidemiological studies (multilevel analysis).

Bayesian spatial modelling is a valuable tool for the geospatial assessment of disease patterns that can help to identify community differences. This method investigates spatial disease patterns and evaluates uncertainty of geographic data. Maps including uncertainty can allow more informed and objective decision-making in relation to targeted disease control, as helping program managers to improve their understanding of decision risk [15,21,22]. Thematic mapping of the 95 percentile range of SIR provides the uncertainty associated with the posterior mean SIR estimates, and identifies LR and HR neighbourhoods with an SIR significantly higher or significantly lower than average risk. On the one hand, most HR neighbourhoods were located in areas for which pulmonary tuberculosis clusters have been detected [7]. The risk was significantly higher in some areas, including the 1^st ^and 4^th ^districts, in which unhygienic conditions prevail. In addition, the neighbourhoods with high posterior mean SIR estimates were those for which the Bayesian credible intervals suggested that pulmonary TB was a potential problem. Objective decisions are required concerning the active detection, prevention or control of TB in these areas. On the other hand, the LR neighbourhoods tended to be located either in the wealthier central neighbourhoods or at the edge of the city. Peripheral neighbourhoods had lower population densities (number of inhabitants/surface area) than those in the city centre. The lower the population density is the less overcrowding should follow resulting hence in an increase of contact distances and thus in decrease of TB transmission. However, the density data may not be reliable because most of this area is uninhabitable (rice fields, swamp), resulting in an underestimation of the true population density. Occupancy rates such as the number of persons per room used for habitation and the percentage of households occupying only one room may be more suitable indicators in developing countries [23]. Furthermore, the detection of neighbourhoods associated with a significantly lower risk should be interpreted with caution, as TB detection is passive in Madagascar and the true rate is not actually known. TB treatments are free in Madagascar so unwillingness to seek medical care probably reflects differences in educational level rather than the financial accessibility of health care. The SIR may also be overestimated for neighbourhoods close to health care facilities, but our results suggested that the positive association between TB incidence rates and the distal variable distance from the patient's residence to the DTC was not significant.

Further data collection is required for neighbourhoods with a low degree of certainty. Unmeasured local factors (e.g., explicit socio-economic factors) not included in the analysis may have a stronger influence in these areas, as TB is associated with socio-economic status. Socio-economic data for all neighbourhoods was not available.

At the district level (ensemble of neighbourhoods), TB incidence rates were found heterogeneous for all districts except the 2^nd ^and 6^th ^districts. The incidence rate in the 2^nd ^district was low. These differences may reflect the underlying heterogeneous spatial distribution of socio-economic status. Uncertainty levels were high for all neighbourhoods in the 6^th ^district, in the north-east part of the city; the least populated area district, in consistency with findings in the US [8]. The greater uncertainty associated with peripheral neighbourhoods may be accounted for by a lack of support from neighbours. Future studies should include guard areas, external to the study area to compensate for edge effects [24].

The geographic patterns seen in these maps may have been influenced by two factors: the number of patients lost to follow-up and the number of households with more than one case. The positive association between households with more than one case and TB risk has been reported in the same setting [7] and elsewhere [13]. Patients lost to follow-up remained contagious and continued to spread the bacillus, resulting in an increase in the risk of TB in their neighbourhoods. The loss of large number of patients to follow-up may reflect low levels of care or a poor follow-up system. As an additional check, positive associations between TB incidence rates and the two significant explicative variables or indicators were confirmed again using a simple linear regression as shown in Figure 3 (as expected, the obtained values of regression coefficients are different from ones in Table 1). Similar checks conducted for the relation between TB incidence and the retreatment, relapse and treatment failure turn out all not statistically significant. The results of our analysis highlight defects in the health care system in low-income country. In these areas, the functioning of the health care system is impaired by diverse factors, including the poor motivation of health workers, cumbersome organisational structures and institutional and personal values.

The spatial scan statistic used in previous studies for determining the spatial distribution of TB has been shown to be complementary to Bayesian methods. The spatial scan approach encompassed many neighbourhoods and tended to detect larger clusters than expected, because the surrounding regions were absorbed, generating false-positives areas due to a lack of specificity; by contrast Bayesian methods minimise false positive rates when used to identify risk areas [17,25,26]. The scan statistic detects general regions in which the risk is significantly high and the Bayesian posterior distribution helps to identify the neighbourhoods contributing strongly to the scan statistic circle [8]. Thus, results for cluster analysis should be interpreted with knowledge of the spatial rate distribution, such as spatial Bayesian rates in particular [27].

## Conclusion

Studies of this type, using a Bayesian approach to estimate both the contribution of variables or indicators related to the health system and the spatial pattern of TB, should be encouraged in epidemiology. Our results confirmed the spatial heterogeneity of TB distribution, with clustering in particular areas observed in Antananarivo, and the importance of covariate effects. The number of households with more than one case is an indicator of TB transmission whereas the number of patients lost to follow up could reflect the efficacy of the health care system for patient follow-up. All the indicators studied here were selected with the aim of ensuring reproducibility of the model, as all were recorded at the DTC and had already been incorporated into the information system routinely used by the TB control program. However, demographic and habitat censuses should be carried out to obtain socio-economic data for neighbourhoods, as the occurrence of TB is known to be linked to both the quality of health care and socio-economic status. The confirmation of TB cluster areas in Antananarivo may help public health authorities to set up priorities regarding to be targeted for prevention or control measures.

## Abbreviations

TB: Tuberculosis; GLMM: Generalized Linear Mixed Model; DTC: Diagnostic and Treatment Centres; CHU: Centre Hospitalier Universitaire (university hospital); DOA: Development Office of Antananarivo; SIR: Standardized Incidence Ratio; CI: Credibility Interval; DIC: Deviance Information Criterion; LR neighbourhoods: low risk neighbourhoods; HR neighbourhoods: high risk neighbourhoods

## Competing interests

The authors declare that they have no competing interests.

## Authors' contributions

RVR made substantial contributions to the concept and design of the study, and helped to analyse and interpret the data and to draft the manuscript. PS was involved in the design of the study, data interpretation, and drafting the manuscript. FR and VR contributed to the design of the study and data interpretation. DJB designed the analytic strategy of the study, interpreted data, drafted the manuscript and revised it critically for major intellectual content. All authors were involved in reviewing all drafts of the manuscript.

## Pre-publication history

The pre-publication history for this paper can be accessed here:

http://www.biomedcentral.com/1471-2334/10/21/prepub
